# Role of the Ubiquitin System in Chronic Pain

**DOI:** 10.3389/fnmol.2021.674914

**Published:** 2021-05-28

**Authors:** Jiurong Cheng, Yingdong Deng, Jun Zhou

**Affiliations:** Department of Anesthesiology, The Third Affiliated Hospital of Southern Medical University, Guangzhou, China

**Keywords:** chronic pain, deubiquitinases, SUMOylation, ubiquitination, ubiquitin ligase

## Abstract

As a significant public health issue, chronic pain, mainly neuropathic pain (NP) and inflammatory pain, has a severe impact. The underlying mechanisms of chronic pain are enigmatic at present. The roles of ubiquitin have been demonstrated in various physiological and pathological conditions and underscore its potential as therapeutic targets. The dysfunction of the component of the ubiquitin system that occurs during chronic pain is rapidly being discovered. These results provide insight into potential molecular mechanisms of chronic pain. Chronic pain is regulated by ubiquitination, SUMOylation, ubiquitin ligase, and deubiquitinating enzyme (DUB), etc. Insight into the mechanism of the ubiquitin system regulating chronic pain might contribute to relevant therapeutic targets and the development of novel analgesics.

## Introduction

Chronic pain is a compounded problem that lasts or recurs for more than 3 months, which significantly affects physical and psychological health (Treede et al., 2019). It is known that chronic pain can result from many pain syndromes. However, the mechanisms underlying chronic pain are complex and not clarified. Therefore, despite a major long-standing investigation for years, there are no effective therapies for most types of chronic pain. Several recent studies have found that ubiquitin system failure is implicated in chronic pain (Tai and Schuman, 2008), mainly including neuropathic pain (NP) and inflammatory pain (Chen et al., 2011). NP is the pain caused by a lesion or somatosensory system disease (Jensen et al., 2011). Inflammatory pain is caused by an increase in the excitability of peripheral nociceptive fibers due to changes in ion channel activity caused by inflammatory mediators (Linley et al., 2010). During the inflammatory process, the pain is triggered by normally innocuous stimuli and becomes chronic if the inflammation is not resolved. The mechanisms of NP are partly distinct from those of inflammatory pain, and potential therapeutic targets mediated by the ubiquitin system to NP and inflammatory pain are different.

The ubiquitin system is a protein degradation pathway mainly composed of ubiquitin, E1 ubiquitin-activating enzyme, E2 ubiquitin-conjugating enzyme, E3 ubiquitin ligase, deubiquitinating enzyme (DUB), and proteasome, etc. The ubiquitination of proteins is a multistep. First, the E1 activates ubiquitin, which is then transferred to E2. Subsequently, the E2∼Ub subsequently interacts with E3. And then, the E3 ligase transfers the E2-bound ubiquitin to a substrate and mediates the ubiquitination (Pickart, 2001). Interaction with the substrate can be direct or *via* ancillary proteins (Glickman and Ciechanover, 2002), finally leading to the degradation of these substrates by proteasomes. Besides, ubiquitination can be antagonized by DUBs, which remove or trim ubiquitin chains on their substrates (Nakamura, 2018). In addition, studies indicate that the ubiquitin–proteasome system (UPS) influences disease onset and progress through the timely degradation of various regulatory proteins (Yi and Ehlers, 2007). Hence, a comprehensive understanding of how the ubiquitin system affects chronic pain might be essential for novel therapeutic opportunities for patients.

## Ubiquitination in Animal Models of Chronic Pain

The pathogenesis of chronic pain involves many different mechanisms and the etiology is multifactorial. Thus, animal models are essential for exploring molecular mechanisms of chronic pain. Peripheral or central nerve injury is commonly used to induce NP. The common models of NP include spinal nerve ligation (SNL) model, sciatic nerve chronic constriction injury (CCI) model, and spared nerve injury (SNI) model (Kumar et al., 2018), while the formalin-induced and complete Freund’s adjuvant (CFA)-induced models are common in inflammatory pain (Muley et al., 2016). Reliable animal models can help understand the mechanisms of the ubiquitin system in chronic pain to develop effective therapeutics.

It is possible that the chronic pain observed in various rodent pain models could be due to an altered ubiquitin system function. Recent studies investigated the expression and function of E3 ubiquitin ligase, deubiquitinase ubiquitin-specific protease 5 (USP5), and Cav3.2 interaction in CCI-triggered NP (García-Caballero et al., 2014). The researchers study the influence of protein ubiquitination on the development of NP and determine mechanical nociception in the pathophysiological processes of NP using a well-characterized rat model of SNL (Lin et al., 2015; Lai et al., 2016, 2018; Liu et al., 2019). SUMOylation disorder after the injury of peripheral nerves, caused by SNI, may alter the Cav3.2 channel activity (Garcia-Caballero et al., 2019; Liu et al., 2019; Tomita et al., 2019). Both formalin and CFA-induced models are valuable experimental methods for inflammatory pain, which provides an empirical basis for understanding the mechanism of the ubiquitin system on inflammatory pain. Altogether, these data indicate that the ubiquitin system is a major determinant of pain response in both inflammatory pain and NP models. Common ubiquitination in animal models of chronic pain is shown in Table 1.

**TABLE 1:** Ubiquitination in animal models of chronic pain.

**Animal models**	**Types of pain**	**The characteristics**	**Ubiquitination in models**	**References**
CCI	NP	Produces unilateral peripheral mononeuropathy associated with nerve compression and neuroinflammatory lesions	Investigate the function of E3 ubiquitin ligase, deubiquitinase USP5	García-Caballero et al., 2014; Lai et al., 2018
SNL	NP	More extensive surgery, injury, and separation of intact spinal segments	Study the influence of protein ubiquitination on the development of NP	Lin et al., 2015; Lai et al., 2016; Liu et al., 2019; Tomita et al., 2019
SNI	NP	Much easier independent of the sympathetic system and last for several weeks,	Used to study drugs associated with USP	Caterina et al., 1997; Garcia-Caballero et al., 2019; Huang et al., 2020
Formalin and CFA model	Inflammatory Pain	Simulate inflammatory lesions be used as a disease model of arthritis	Understand the mechanism of the ubiquitin system	Cottrell et al., 2006

*Sciatic nerve injury of the peripheral nerve is often used to induce neuropathic pain (NP). Formalin and complete complete Freund’s adjuvant (CFA) often induce chronic inflammatory pain. Animal models may help to understand the symptoms and physiology of related pain. Several animal models help in understanding the mechanism by which the ubiquitin system regulates chronic pain.*

## Role of the Ubiquitin System in Neuropathic Pain

### Mechanism of Ubiquitination in Neuropathic Pain

Protein ubiquitination plays a crucial role in the development of NP (Lai et al., 2016). This function is achieved by ubiquitination-modified protein receptors and ion channels to affect synaptic activity and efficiency.

NP associated with ubiquitination partly works by regulating signal transduction pathways. The transient receptor potential vanilloid-1 (TRPV1), a member of the transient receptor potential (TRP) family, is considered a therapeutic target for pain relief (Caterina et al., 1997). TRPV1 promotes the ubiquitination of epidermal growth factor receptor (EGFR) in cells and regulates EGFR/mitogen-activated protein kinase (MAPK) signaling through the lysosomal degradation pathway, leading to increased cytoplasmic translocation and degradation of EGFR, and then downregulation of EGFR level. It responds to harmful stimuli from afferent nerve terminals and participates in pain and inflammation (Huang et al., 2020). Lysine ubiquitination is a signal for the transport and degradation of G protein-coupled receptors. Short-term stimulation of substance P (SP) can induce the endocytosis and circulation of the neurokinin-1 receptor (NK1R). Chronic stimulation of SP induces the ubiquitination of lysine residues in NK1R cells, which mediates its degradation and downregulation. In this process, tachykinin is released continuously and prevents nociceptive signals (Cottrell et al., 2006). In addition, tumor necrosis factor-alpha (TNF-α) influences the development of NP through the ring finger protein (RNF20)/histone H2B monoubiquitination (H2Bub)/RNA polymerase II (pRNAPII)/metabotropic glutamate receptors (mGluR5) signal transduction cascade. Specifically, TNF-α-induced RNF20-driven H2B monoubiquitination can promote dorsal horn phosphorylated RNAPII-dependent mGluR5 transcription, affecting protein ubiquitination and degradation (Lai et al., 2016). Tumor necrosis factor receptor-associated factor 2 (TRAF2) and NcK-interacting kinase (TNIK) are enhanced, and the TNIK is coupled to glutamate receptor (GluR1) after nerve injury. TRAF2 is regulated by F-box protein 3 (Fbxo3) and ubiquitination of leucine-rich repeat protein 2 (Fbxl2). Thus, Fbxo3-dependent Fbxl2 ubiquitination and degradation participate in the development of NP by upregulating TRAF2/TNIK/GluR1 signaling (Lin et al., 2015). Studies have demonstrated that TRAF6 has E3 ligase activity; the inhibition of TRAF6 expression can effectively relieve SNL-induced NP. Taken together, the results suggest that TRAF6 serves an important role in NP progression and pathogenesis; the exact mechanism remains to be elucidated (Dou et al., 2018).

The other part of ubiquitination mediates the NP process by modifying the activity of synaptic proteins and ion channels. Studies on low voltage-gated calcium channels suggest that Ca^2+^ elevation in sensory neurons is associated with NP (Bourinet et al., 2014; François et al., 2014). Rab3-interactive molecule (RIM) can be associated with N-type voltage-dependent calcium channels. Rab3-interactive molecule-1α (RIM1α) upregulates the expression of CaV2.2 by recruiting and coupling with CaV2.2. This enables the rapid and synchronized release of neurotransmitters at the presynaptic site to mediate NP. Other studies have demonstrated that Fbxo3 affects the activity of CaV2.2 by inhibiting fbxl2-dependent RIM1 ubiquitination. Moreover, Mas-related G-protein-coupled receptor subtype C (MrgC) ubiquitination affects RIM1α/Ca_*V*_2.2 cascade and then reduces intracellular calcium concentration to participate in the occurrence and maintenance of chronic pain (Marangoudakis et al., 2012; Lai et al., 2016; Sun et al., 2019).

Ubiquitination can also regulate NP through nociceptive information modulation and synaptic function regulation in nociceptive neurons (Kiris et al., 2014). TNF-α impedes presynaptic active zone protein (Munc13-1) ubiquitination in the spinal cord, which promotes the synaptic activity of nociceptive neurotransmission in NP (Lindenlaub et al., 2000; Hsieh et al., 2018). The reduction of ubiquitination of transporter proteins induces the upregulation of glycine transporter 2 (GlyT2) activity, which affects noxious signal conduction (Villarejo-López et al., 2017). Experiments have shown that periaqueductal gray matter (PGM) in the midbrain, which is involved in pain processing and regulation, could be overexcited and dysfunctional in the face of stress. This process is accompanied by the activation of the ubiquitination system, which may contribute to excruciating pain (Quan et al., 2005). Some drug researches are also based on the role of ubiquitination in pain. For example, metformin can eliminate the abundance of ubiquitinated proteins, which can achieve analgesia by inhibiting cell apoptosis (Weng et al., 2019). In brief, these results emphasize that inhibition of the ubiquitination process of proteins or ion channels may achieve adequate chronic pain relief. Therefore, we summarized the possible mechanisms and potential targets of the abovementioned ubiquitination process of NP regulation, aiming to provide new ideas and experimental evidence for the application of strategies to promote the treatment of chronic pain by influencing the ubiquitination process.

### SUMOylation and Neuropathic Pain

SUMOylation is a small ubiquitin-like modifier (SUMO) protein family coupled to lysine residues (Yang et al., 2017) and is correlated with ubiquitination. It is an important posttranslational protein modification that participates in NP transmission and modulation (Henley et al., 2014). The members of the collapsin response mediator protein (CRMP) family are an effect of neuronal polarity and synapse dynamics. Dysregulation of CRMP has now been described in numerous diseases (Tan et al., 2014). CRMP2 SUMOylation has been reported as the main factor that mediates chronic pain (Moutal et al., 2019). Studies have shown that CRMP2 SUMOylation can regulate voltage-gated ion channels, especially the sodium channels in the pain signaling process (Dustrude et al., 2013; Baumann and Kursula, 2017; Bennett et al., 2019), which are upregulated to induce NP (Barbosa and Cummins, 2016). Precisely, the combination of CRMP2 and SUMO controls the expression and density of Nav1.7, thus affecting its activity (Dustrude et al., 2016; François-Moutal et al., 2018a; Moutal et al., 2018). In addition, the E2 SUMO conjugating enzyme (Ubc9) is necessary for SUMOylation and interacts with CPMP2 to reduce CRMP2 SUMOylation, which in turn reduces Nav1.7 current to alleviate NP (François-Moutal et al., 2018b; Moutal et al., 2020). Therefore, preventing CRMP2 SUMOylation may be an effective target to reverse NP (Khanna et al., 2020). Thus, further research is necessary before translating preclinical findings into the clinic setting. There are many clinical challenges to developing SUMOylation enzyme inhibitors, notably to determine which specific targets and predictive indicators are targeted, in order to determine what kind of specific mechanisms may be more beneficial.

### Ubiquitin Ligase Modulation of Neuropathic Pain

Ubiquitin ligase is the most critical factor in determining specificity during protein ubiquitination. E3 ubiquitin ligase affects the development of pain by promoting protein ubiquitination and degradation. Since E3 ligase determines the specificity of the reaction, they have attracted the most attention. E3 ubiquitin ligase includes three families: HECT (homologous to the E6AP carboxyl terminus), RING (really interesting new gene), and U-box domains (Zheng and Shabek, 2017).

It is well known that E3 ubiquitin ligase can regulate ion channel protein levels through ubiquitination. The late-promoting complex/RING body, anaphase-promoting complex (APC/C), is a cullin-RING-E3 ubiquitin ligase. Its co-activator (Cdh1) is essential for proliferating cells and terminally differentiated neurons. It has been revealed that downregulation of the Cdh1 signal in spinal dorsal horns contributes to the maintenance of mechanical allodynia after nerve injury in rats; thus, it may upregulate the expression of Cdh1 in the spinal cord to induce pain relief (Hu et al., 2016).

**FIGURE 1:**
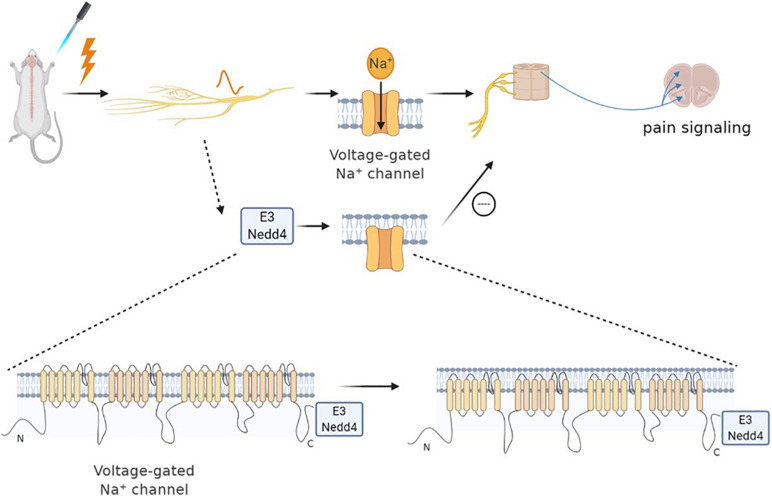
Neural precursor cell expressed developmentally downregulated protein 4 (NEDD4) family of E3 ubiquitin ligase can effectively regulate Nav channels. Sodium channels play an important role in neuropathic pain. When nociceptors are activated, Nav1.7 and Nav1.8 are upregulated, leading to pain. The NEDD4 family of E3 ubiquitin ligase can react on sodium ion channels to ubiquitinate them and promote the channel endocytosis. Specifically, NEDD4 ubiquitin ligase can react with the PY motif at the end of the C subunit of each Nav subtype, thereby regulating the expression of the Nav channel, as well as the current density and neuronal excitability. Therefore, the pain signal of sensory neurons can be reduced indirectly by regulating the activity of NEDD4-2.

Neural precursor cell expressed developmentally downregulated protein 4 (NEDD-4) is a specific E3 ubiquitin ligase regulating N-methyl-D-aspartate (NMDA) receptors (NMDARs) with the GluN2D (NMDAR subtypes) subunit through ubiquitination-dependent downregulation (Goel et al., 2015). Specifically, NMDARs are a family of glutamate-gated ion channels that can regulate various central nervous system (CNS) functions. The hypo- or hyper-activation of NMDARs is intimately associated with certain neurological diseases (such as chronic pain, neurodegenerative diseases). Nedd4 can attenuate NP *via* ubiquitination-dependent downregulation of the NMDAR of the GluN2D subunit (Gautam et al., 2013). Besides, several studies have revealed that the NEDD4 family of E3 ubiquitin ligase can effectively regulate the Nav channels (Eaton et al., 2010). As mentioned earlier, the upregulation of Nav1.7 and Nav1.8 leads to NP. Each Nav subtype has a PY (PPxY) motif at the end of subunit C, making them a target for the NEDD4 ubiquitin ligase family. NEDD-4 like (NEDD4-2) is a ubiquitin-protein ligase that belongs to the NEDD4 family of E3 ubiquitin ligase. Therefore, NEDD4-2 can ubiquitinate Nav1.7 and Nav1.8 and then regulate the ion channel of the cell membrane (Staub et al., 2000). Specifically, NEDD4-2 exists and acts on Nav1.7 and Nav1.8 in dorsal root ganglion (DRG) neurons. When the peripheral nerve is injured, NEDD4-2 in the neuron is downregulated and then disorders Nav, leading to nervous overexcitement and pain (Ekberg et al., 2014; Laedermann et al., 2014). NEDD4-2 regulates nociceptive sensations, and its dysfunction causes NP occurrence. Therefore, active NEDD4-2 can inhibit the upregulation of sodium channels to cure NP (Bongiorno et al., 2011; Cachemaille et al., 2012; Laedermann et al., 2013, 2014; Figure 1).

Cav3.2 channels can be ubiquitinated and are capable of intracellular modification and degradation. The ubiquitination state of Cav3.2 channel in the pain pathway is regulated by the interaction of HECT E3 ligase E3 ubiquitin-protein ligase 1 (WWP1) and USP5, which can regulate the stability of the T-channel protein in the plasma membrane. WWP1 binds to intracellular domains III–IV regions of Cav3.2 T-type and modify specific lysine residues in this region. Preventing the enhancement of this current may potentially combat the development of pain (García-Caballero et al., 2014). The bone morphogenetic protein (BMP) signal downstream of highwire E3 ligase can stimulate nociceptors. The upregulation of the BMP signal leads to a significant increase in Ca^2+^ current, which is based on physiologically sensitized nociceptors and nociceptive behavior (Honjo and Tracey Jr., 2018).

Pellino 1, a critical mediator in various immune receptor signaling pathway molecules, forms the conserved E3 ubiquitin ligase family. It can regulate NP by regulating MAPK/nuclear factor kappa B (NF-κB) signaling in the spinal cord (Börner and Kraus, 2013; Wang et al., 2020). Also, E3 ubiquitin ligase Caritas B cell lymphoma (Cbl) is a highly conserved ubiquitin ligase expressed in both normal and tumor cells. Besides, c-Cbl can relieve pain by inhibiting the activation of spinal microglia and reducing the release of inflammatory factors. A study found that nerve injury could downregulate the expression of c-Cbl, which leads to the activation of microglia, the increase of inflammatory factor release, and the occurrence of NP through the extracellular signal-regulated kinase (ERK) pathway (Chen et al., 2014; Zhang et al., 2019a). Therefore, increasing c-Cbl could relieve NP (Xue et al., 2018). Interestingly, when the expression of E3 ubiquitin ligase Cbl family increases, it reduces the analgesic effect of interleukin 2 (IL-2) by increasing ubiquitination of Zeta-chain associated protein 70 (ZAP70) and phospholipase C-γ1 (PLC-γ1) (Jeong et al., 2018). Thus, ubiquitin and E3 ubiquitin ligases c-Cbl can regulate the development of NP by reducing the production of IL-2. These findings indicate that the ubiquitin E3 ligase mainly decreases NP by modulating ion channel activity and influencing different signaling pathways. This mechanism of action illustrates how the interdependence between E3 ubiquitin ligase and its substrate protein can provide new therapeutic targets. Therefore, the effects of E3 ubiquitin ligase should be considered when preventing the development of neuralgia after nerve injury.

### Role of the Deubiquitinating Enzyme

**FIGURE 2:**
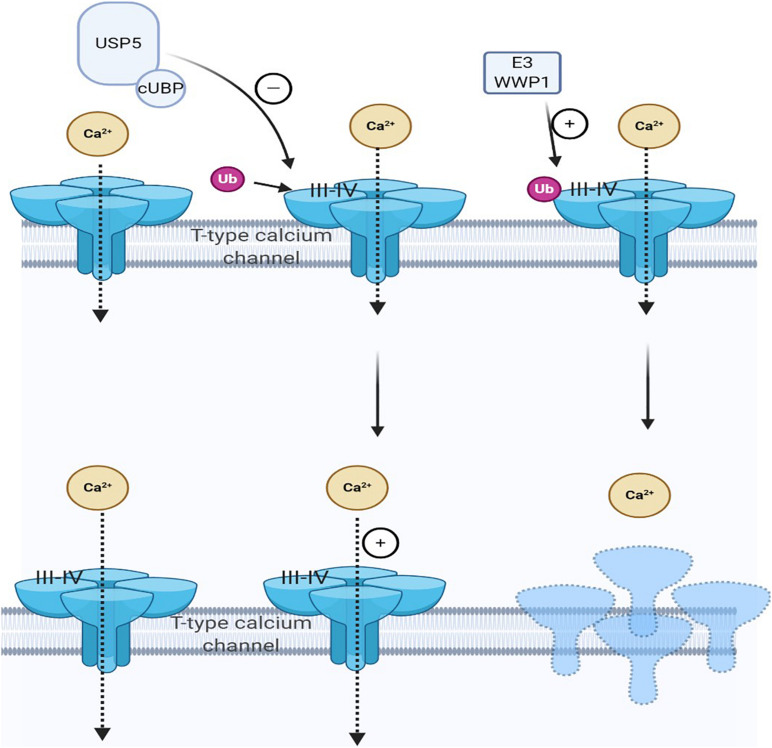
Cav3.2 channel ubiquitination status is regulated by the interaction of E3 ligase and ubiquitin-specific protease 5 (USP5). The ubiquitination state of Cav3.2 channel in the pain pathway is regulated by the interaction of USP5 and HECT E3 ligase HECT E3 ligase E3 ubiquitin-protein ligase 1 (WWP1), which can regulate the stability of the calcium channel protein in the plasma membrane. When pain occurs, USP5 is upregulated and its cUBP domain interacts with the III–IV junction of Cav3.2 T-type calcium channels. This can reduce the ubiquitination of Cav3.2 channels and upregulate Cav3.2 channels, making them more stable on the cell surface. WWP1 also binds to intracellular regions III–IV in the Cav3.2 t-channel junction region and modifies specific lysine residues in this region.

Both deubiquitination and ubiquitination are involved in maintaining cell homeostasis. Ubiquitin chain, mediated by DUB, is vital in various cellular processes. USP is a cysteine deubiquitinase and USP5 is recognized to be explicitly polyubiquitin, which is not bound to the target protein and is involved in pain development (Kowalski and Juo, 2012; Ning et al., 2020). Deubiquitination of the channel mediates Cav3.2 T-type calcium by deubiquitinase USP5. When the nerve is damaged, USP5 enhances the deubiquitination of the Cav3.2 channel in the dorsal horn, increasing T-type calcium current, which in turn causes pain hypersensitivity (Gadotti et al., 2015; Gadotti and Zamponi, 2018; Ozaki et al., 2018). Specifically, USP5 is upregulated in NP. Meanwhile, a specific amino acid region in the zinc-finger ubiquitin-specific protease (cUBP) domain of USP5 interacts with the III–IV linker region of the Cav3.2 T-type calcium channel. The reduction of the ubiquitination of the Cav3.2 channel increases the stability of the Cav3.2 channel at the cell surface. Therefore, through disruption, the interaction between USP5 and Cav3.2 channel inhibits the expression of Cav3.2; hence, inhibiting T-type calcium current may alleviate NP (Garcia-Caballero et al., 2016). Some factors also affect the interaction between USP5 and Cav3.2 channels, such as IL-1 recognition (Stemkowski et al., 2017), USP5 SUMOylation disorder (Garcia-Caballero et al., 2019), and TRPV1 nociceptors (Stemkowski et al., 2016). Therefore, these factors also reduce Cav3.2 channel activity by weakening USP5 regulation and increasing channel ubiquitination to achieve the purpose of analgesia (García-Caballero et al., 2014; Joksimovic et al., 2018; Figure 2). For therapeutic purposes, interference with USP5/t-type channel interaction will specifically target a process involving abnormal upregulation of channel activity while maintaining normal channel function, thereby reducing the risk of adverse side effects.

Another deubiquitination enzyme, ubiquitin C-terminal hydrolase L1 (UCHL1), is crucial in neurological diseases. As the UCH-L1 activity increases, ubiquitin expression is upregulated. Extracellular ubiquitin is an agonist of CXC motif chemokine receptor type 4 (CXCR4), which is involved in several pathological conditions, such as immunologic, oncologic, and neurologic disorders. Ubiquitin and CXCR4 may lead to microglial activation and NP. Thus, inhibiting spinal cord UCHL1 and suppressing ubiquitin expression and microglial activation may be effective (Cheng et al., 2016). Although there is limited information on DUBs in published early preclinical trials, some information can be emphasized. DUB can regulate the stability and degradation of proteins through a variety of cellular pathways. Here, we present evidence that DUBs can act as a potent regulator of chronic pain processing by increasing the stability of the T-channel or promoting the activation of microglia. This may provide a theoretical basis for the development of more effective chronic pain therapeutics.

### Ubiquitin–Proteasome System in Neuropathic Pain

**FIGURE 3:**
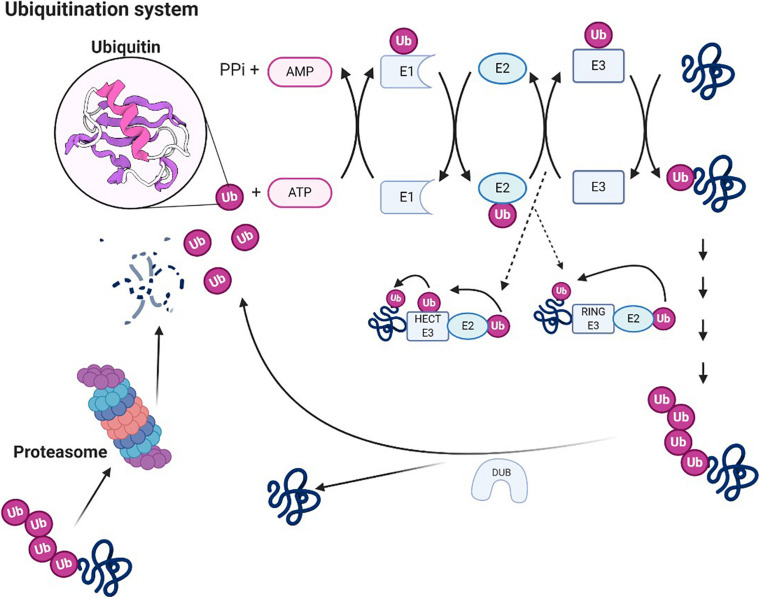
Ubiquitin system. Ubiquitin molecules can be covalently linked to other substrates to form ubiquitin chains. A chain of four or more ubiquitin units constitutes proteasome 26S. Ubiquitin is an ATP-dependent process catalyzed by three enzymes (E1, E2, and E3), which also confer specificity to the process. The first reaction catalyzed by E1 is responsible for the activation of ubiquitin. E1 transfers ubiquitin on cysteine to E2. Through the activity of transferase, E2 transfers the ubiquitin to E3 ligase (two groups of protein ubiquitin ligase, HECT E3 enzymes and ring E3s, the way they transfer ubiquitin is exactly different), which binds ubiquitin to the Lys residue of the target protein. Polyubiquitinated protein can be degraded by 26S proteasome, and ubiquitin can be recovered under the action of the deubiquitinating enzyme.

UPS is a selective non-lysosomal proteolytic system in which substrates are labeled with ubiquitin and can be degraded by proteasomes (Ji and Kwon, 2017; Caputi et al., 2019). The necessary step in the degradation pathway of the proteasome is the formation of ubiquitin–protein conjugates. Covalent binding of ubiquitin ligase and its target molecule leads to molecular degradation. This system can degrade various cellular proteins that can regulate cell growth or function (Figure 3). UPS affects NP by degrading several proteins essential to synaptic plasticity; its downregulation can effectively relieve pain (Hegde, 2010). UPS activity increases during nerve injury, leading to hyperalgesia. Proteasome inhibitors can prevent and reverse NP by concentrating protein systems, including protein systems that control the release of dynorphin A and calcitonin gene-related peptide (CGRP) and the postsynaptic effect of dynorphin A (Moss et al., 2002; Ossipov et al., 2007). Recent studies suggest that reducing proteasome degradation of Cav3.2 T-type calcium channels is associated with persistent pain. When proteasome degradation is inhibited, which subsequently inhibits the upregulation of USP5, the protein level of Cav3.2 in nociceptors is increased (Tomita et al., 2019). In addition, when the UPS was activated, the degradation of glutamate transporters is enhanced, resulting in mechanical hyperalgesia (Manning, 2004; Zavrski et al., 2005; Yang et al., 2008; Stockton Jr., Gomes et al., 2015). Accordingly, inhibition of ubiquitin proteasome-mediated degradation of glutamate transporters may offer treatment options for certain neurological diseases and chronic pain. While this methodology deserves further investigation, considering the diversified effects of the proteasome, the use of broad-spectrum proteasome inhibitors may be flawed, and the development of selective inhibitors for chronic pain may be a more effective method. In this sense, developing methods for reducing chronic pain with selective inhibitors could be a more effective approach.

## Role of the Ubiquitin System in Inflammatory Pain

The etiology and pathogenesis of NP and inflammatory pain remain inconsistent, and their targets in the ubiquitin system are also different. Therefore, novel therapeutic targets for chronic pain states accompanying inflammatory processes are urgently needed. As described below, inflammatory disease models show the relief of inflammatory pain by inhibiting the ubiquitination process of several proteins. Therefore, it is necessary to explore the role of the ubiquitin system that regulates pain signals during chronic inflammation in order to reflect the mediated analgesia in different forms of chronic pain.

### Ubiquitination in Inflammatory Pain

Ubiquitination is significant in inflammatory pain. Acute inflammation is a key player in peripheral sensitization and local tissue inflammation-evoked pain. The pain persists and becomes chronic during inflammation resolution. Short-term stimulation of SP induces TRPV1 polyubiquitination, which has an impact on chronic visceral pain. Various pro-inflammatory mediators, such as histamine and bradykinin, express function by regulating TRPV1. The degree of abdominal pain in patients is related to the increased expression of TRPV1. The feedback regulation of TRPV1 by SP also participates in the inflammatory visceral pain. Therefore, drugs that block SP-mediated TRPV1 ubiquitination may reduce chronic pain after inflammation (Lapointe et al., 2015).

The inhibition of glycine is weakened after peripheral tissue damage, which is considered a critical factor in the occurrence of inflammatory pain. The inhibition of glycine can increase the excitability and spontaneous activity of spinal cord nociceptive neurons during inflammatory pain. Studies found that the activity of the glycine receptor subunit (GlyRs-α1) is dependent on ubiquitination, which may help glycinergic inhibition after peripheral inflammation. Therefore, ubiquitin modification of Glyrs-α1 reduces the spinal glycinergic inhibition in peripheral inflammation. Furthermore, preventing Glyrs-α1 from ubiquitination, restoring Glyrs-α1-mediated synaptic transmission can generate analgesic action (Zhang et al., 2019b). Despite these studies further clarifying the role of ubiquitination in inflammatory pain, more preclinical trials are still necessary. The development of new drugs targeting specific mechanisms may facilitate translation of these findings from bench to bedside.

### Role of the Deubiquitinating Enzyme in Inflammatory Pain

As mentioned earlier, USP5 also mediates inflammatory pain by enhancing the Cav3.2 channel activity. Inflammatory mediators, bradykinin, also increase the number of sensory neurons expressing T-type Ca^2+^ channels (Huang et al., 2016; Sekiguchi et al., 2018). USP5 binds to III–IV linkers in Cav3.2 (Garcia-Caballero et al., 2016). Cav3.2 channel ubiquitination is the substrate of USP5, and its association with USP5 leads to changes in Cav3.2 protein level and current density. USP5-mediated regulation of Cav3.2 is abnormally enhanced in chronic pain. These data suggest that USP5 is a regulator of chronic pain associated with inflammatory processes (García-Caballero et al., 2014).

Ubiquitin-specific proteases or deubiquitin enzymes remove ubiquitin groups from the degraded target proteins, thereby improving protein stability. Therefore, USP5 knockout by shRNA can increase Cav3.2 ubiquitination, decrease Cav3.2 protein level, and decrease calcium current. It was found that inhibition of USP5 *in vivo* or decoupling of USP5 from the intrinsic Cav3.2 channel through intrathecal delivery of Tat peptide can protect from inflammatory pain. Therefore, targeting Cav3.2 deubiquitination by USP5 is a potential target for pain conditions associated with inflammatory disorders (Gadotti et al., 2015). According to data from basic research, combined with experimental pain models, the method of inhibiting DUBs has been studied in preclinical studies. It can be concluded that the mechanisms listed above contribute to relieve chronic pain, especially inflammatory pain.

### Ubiquitin Ligase Modulation of Inflammatory Pain

E3 ubiquitin ligase is a key component that generates specific reactions through substrate recognition (Qiu et al., 2011). The substrates of NEDD4-2 mainly include the epithelial sodium channel (ENaC) and neurotrophin receptor (TrkA). NEDD4-2 heterozygous mice can provide a new model for studying inflammatory pain, which causes the mice to be more hyperactive and increase their sensitivity to pain during central sensitization and inflammation. This is likely to be caused by decreased levels of NEDD4-2, leading to increased reactivity of the substrate TrkA to nerve growth factor (NGF). Therefore, a full complement of E3 ubiquitin ligase is necessary for inflammatory pain relief (Yanpallewar et al., 2016).

Ubiquitin protein ligase E3 component n-recognin 5 (UBR5) is a kind of HECT (homologous to E6AP C-terminus), which can recognize another E3 ubiquitin ligase of N-Degrons. It has the catalytic ability to directly identify and link ubiquitin to protein degradation. UBR5 can regulate neuronal plasticity by activating NMDARs in the CNS. It participates in the pathological process of CNS diseases through modified ubiquitination, which is crucial in regulating nociception (Pierre et al., 2008). This adjustment is mainly through the subsequent process. Additionally, for CFA-induced chronic inflammatory pain, the increased expression of circRNA-Filip11 (serine A interacting protein 1-like) in the spinal cord regulates nociception by UBR5. The downregulation of UBR5 significantly reduced the nociceptive response induced by Lenti-Filip11. miRNA-1224-mediated splicing of circRNA-Filip11 regulates chronic inflammatory pain throughout by targeting UBR5. Therefore, the development of inflammatory pain could be reduced by inhibiting UBR5 (Pan et al., 2019).

Myc binding protein 2 (MYCBP2) is another E3 ubiquitin ligase, which can inhibit neuron growth and synapse formation by regulating various signaling pathways. Research has proven that its selective deletion in macrophages can reduce zymogen-induced inflammatory pain and promote the resolution of inflammation (Pierre et al., 2018). Other E3 ubiquitin ligases, such as ligand of numb proteins X1/2 (LNX1/LNX2), are functional regulators of neuronal GlyT2. LNX1 and LNX2 interact with GlyT2 and ubiquitinate the C-terminal cluster of the transporter lysine to control the expression and activity of GlyT2. Changes in the expression or activity of GlyT2 lead to the emptying of synaptic vesicles, which may be related to inflammatory pain pathology (de la Rocha-Munoz et al., 2019). Furthermore, the phosphorylation of E3 ubiquitin ligase Cbl-b weakens its binding and ubiquitination to GLUN2B (a type of NMDAR subunit), thereby inhibiting GLUN2B-mediated synaptic currents and inflammatory pain (Zhang et al., 2020).

HUWE1 (HECT, UBA, and WWE domain contains 1), an E3 ubiquitin ligase located in the spinal cord synapse, specifically interacts with GlyRs-α1. It can reduce the surface expression of GlyRs-α1 by ubiquitinating GlyRs-α1. Ubiquitin modification of GlyRs-α1 is a crucial way to reduce peripheral inflammation inhibited by spinal cord glycine. After the surrounding tissues are injured, the inhibitory effect of glycine is weakened, which leads to the occurrence of inflammatory pain. Previous studies showed that HUWE1 contributed to glycinergic disinhibition. Additionally, knockout of HUWE1 can attenuate GlyRs-α1 ubiquitination, improve glycinergic synaptic transmission, and reduce inflammatory pain. Therefore, it can interfere with the activity of HUWE1and produce analgesic effects by restoring GlyRs-α1-mediated synaptic transmission (Zhang et al., 2019b). This E3-ubiquitin ligase-mediated protein ubiquitination regulates chronic inflammatory pain *via* controlling the level of substrate proteins and then possibly adjusts synaptic efficacy. The results will offer a basis for future research on the role of protein ubiquitination in inflammatory pain (Table 2).

**TABLE 2:** Available potential targets of E3 ubiquitin ligases and DUBs for chronic pain.

**Categories**	**The names of the enzymes**	**Targets**	**Pain relief**	**Types of chronic pain**	**References**
E3 ubiquitin ligases	WWP1	Cav3.2	↑	NP	García-Caballero et al., 2014
	Fbxo3	TRAF2/TNIK/GluR1 Cascade	↓	NP	Lai et al., 2016
	Fbxo45	Munc13-1	↓	NP	Lindenlaub et al., 2000
	APC/C	Cdh1	↑	NP	Goel et al., 2015
	NEDD4-2	GluN2D	↑	NP	Staub et al., 2000; Eaton et al., 2010
		Nav1.7, Nav1.8	↑		
	Peli1	MAPK/NF-κB signal	↓	NP	(Börner and Kraus, 2013
	c-Cbl	microglia	↑	NP	Kowalski and Juo, 2012
		ZAP70, PLC-γ1	↓		
	HUWE1	GlyRs-α1	↓	Inflammatory pain	Sekiguchi et al., 2018
	UBR5	circRNA-Filip11	↓	Inflammatory pain	Pierre et al., 2018
DUBs	USP5	Cav3.2	↓	NP, Inflammatory pain	García-Caballero et al., 2014
	UCHL1	Microglial	↓	NP	Ji and Kwon, 2017

*↑ Represents upregulation of the enzymes can relieve pain.*

*↓ Represents downregulation of the enzymes can relieve pain.*

*According to the role of the E3 ubiquitin ligases and DUB in chronic pain. Summarized their targets and specific roles in the pain process. APC/C, anaphase-promoting complex; DUB, deubiquitinating enzymes; Fbxo, F-box protein; HUWE1, HECT, UBA, and WWE domain contains 1; MAPK, mitogen-activated protein kinase; NEDD4, neural precursor cell expressed developmentally downregulated protein 4; NF-κB, nuclear factor kappa B; NIK, NcK-interacting kinase; NP, neuropathic pain; TRAF2, tumor necrosis factor receptor-associated factor 2; UBR5, ubiquitin protein ligase E3 component n-recognin 5; UCHL1, ubiquitin C-terminal hydrolase L1; USP5, ubiquitin-specific protease 5; WWP1, HECT E3 ligase E3 ubiquitin-protein ligase 1.*

### Ubiquitin–Proteasome System in Inflammatory Pain

UPS is a key intracellular regulator of inflammation and pathological pain. Intracellular pathways mediated by UPS affect inflammatory pain. It can affect the expression of sensory neuropeptides, which can regulate pain and inflammation.

UPS inhibitors can prevent inflammatory pain. Proteasome inhibitor MG132 reduces inflammatory pain by targeting the sensory neuropeptide SP of the CNS in arthritis. Specifically, the NF-κB family is a major modulator of immune and inflammatory processes following injury and infection (Napetschnig and Wu, 2013). MG132 can inhibit the activation of NF-κB to reverse the inflammatory pain (Ahmed et al., 2017). The generation of new proteasome inhibitors may represent a new pharmacotherapy for inflammatory pain.

Whether the application of a wide range of proteasome inhibitors may have complex effects in these ubiquitination pathways, even limited to the nervous system, and whether it causes adverse effects require careful consideration.

## Perspectives and Conclusion

A large body of evidence has provided a partial explanation of how changes in the ubiquitin system result in improved chronic pain response.

The examples presented here provide only a glimpse into the expanding role of ubiquitination in regulating chronic pain. The ubiquitin system is essential not only in the process of regulating chronic pain but also in pain treatment. However, understanding the molecular mechanisms underlying pain regulation by ubiquitin remains challenging because more components of the ubiquitin system are linked to chronic pain, such as ubiquitin ligase, UPS, and DUBs. And the differences between NP and inflammatory pain are not reported to be specific in ubiquitination. The underlying specific difference needs to be revealed in future studies.

In the process of pain, the ubiquitin signaling pathway often acts in cascade with other nociceptors, which further complicates chronic pain. Dysregulation of gene expression mediated by epigenetic mechanisms is crucial in the occurrence and maintenance of chronic pain caused by multiple reasons. To understand the dynamics and complexity of such events, it is necessary to conduct comprehensive proteomic and genomic research using various types of chronic pain models.

Understanding how the ubiquitin system affects the development of chronic pain can provide new ideas and strategies for pain treatment. Interfering with the ubiquitin pathway using inhibitors, such as proteasome inhibitors, is an effective strategy to treat chronic pain. The application of these strategies in clinical trials will expand and diversify the scope of chronic pain treatment for more effective therapies of patients in the future. Therefore, a deeper insight into the ubiquitin system will be precious for the future development of chronic pain therapies.

## Author Contributions

JC, YD, and JZ contributed to conception and design of the study. JC wrote the first draft of the manuscript. YD accessed data. JZ contributed to manuscript revision. All authors contributed to the article and approved the submitted version.

## Conflict of Interest

The authors declare that the research was conducted in the absence of any commercial or financial relationships that could be construed as a potential conflict of interest.
